# EXAMINING THE SOCIAL AND TECHNOLOGICAL BENEFITS OF AN INTERGENERATIONAL PROGRAM FOR OLDER ADULTS

**DOI:** 10.1093/geroni/igz038.551

**Published:** 2019-11-08

**Authors:** Skye N Leedahl, Itza Serrano

**Affiliations:** University of Rhode Island, Kingston, Rhode Island, United States

## Abstract

Intergenerational programs are increasingly being implemented across the country. Much of the research on these programs has shown beneficial outcomes for students related to reductions in aging stereotypes, but less in-depth research has been done examining older adult outcomes (see Andreoletti & Howard, 2016 for a review). Currently, faculty and students from the University of Rhode Island (URI) are implementing the Engaging Generations: Cyber-Seniors Program, which connects older adults with student mentors to provide technological support to older adults, help students build communication and teaching skills, and overall build positive intergenerational relations. The aim of this study was to evaluate the outcomes of intergenerational programming participation for older adults (N=41) related to social isolation, loneliness, social engagement, and digital competency, measured using pre and post surveys given to participants. SPSS software was used to conduct descriptive analysis, t Tests, and one-way ANOVAs. Thematic analysis was used to analyze open-ended participant responses. Results showed significant improvements on items of the digital competency scale, particularly in relation to social media and for those who started with lower levels of digital competence, but not for other measures. Qualitative analysis showed that the older adults valued the technological knowledge gained, and the pleasant interactions and pedagogy. This study advances the engagement theory principle of problem-based learning. Much can be learned from both the significant and non-significant results to help inform intergenerational programming and technology support programs for older adults.

